# A proposal for the future of medical education accreditation in Korea

**DOI:** 10.3352/jeehp.2020.17.32

**Published:** 2020-10-21

**Authors:** Ki-Young Lim

**Affiliations:** Department of Psychiatry and Behavioral Sciences, Ajou University School of Medicine, Suwon, Korea; Hallym University, Korea

**Keywords:** Accreditation, Graduate medical education, Medical education, Republic of Korea, Self-assessment

## Abstract

For the past 20 years, the medical education accreditation program of the Korean Institute of Medical Education and Evaluation (KIMEE) has contributed significantly to the standardization and improvement of the quality of basic medical education in Korea. It should now contribute to establishing and promoting the future of medical education. The Accreditation Standards of KIMEE 2019 (ASK2019) have been adopted since 2019, with the goal of achieving world-class medical education by applying a learner-centered curriculum using a continuum framework for the 3 phases of formal medical education: basic medical education, postgraduate medical education, and continuing professional development. ASK2019 will also be able to promote medical education that meets community needs and employs systematic assessments throughout the education process. These are important changes that can be used to gauge the future of the medical education accreditation system. Furthermore, globalization, inter-professional education, health systems science, and regular self-assessment systems are emerging as essential topics for the future of medical education. It is time for the medical education accreditation system in Korea to observe and adopt new trends in global medical education.


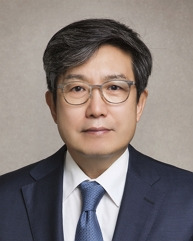


## Introduction

Twenty years have passed since the 1st basic medical education accreditation project was launched for the medical education system in Korea. For the past 20 years, this accreditation system has prevented the indiscriminate establishment of medical schools and has enabled the structural standardization of educational resources and the curriculum, including medical facilities and teaching personnel. This system has also made countless contributions to the advancement of Korean medical education, including the introduction of an innovative curriculum, the establishment of humanities and social medicine education, and the spread of performance-based education and student-centered education. The present study aimed to suggest directions that the Korean medical education accreditation project, which has entered into its adulthood at the age of 20 years, should pursue in the future.

## Beginning of Korea’s medical education accreditation system

Medical school accreditation was first discussed in Korea in 1990, in an article titled “Need for medical school assessment system” which was published in the second volume of the *Korean Journal of Medical Education*. Professor Yoo Bok Lee of the Yonsei University College of Medicine argued persuasively for the need to introduce an accreditation system as the best way to ensure the quality of medical education [1]. The background of these claims was the sudden and unilateral turmoil introduced by new medical schools. He articulated this point as follows:

Since medical education has the longest history and tradition in the field of higher education in our country, it has been a matter of pride to advance and improve medical education in many ways. However, it is true that in the past 20 years, the quantitative expansion of medical education institutions and the rapid increase in the number of students have instead caused the deterioration of education. Therefore, the biggest task facing medical education in Korea is the structural improvement of medical education institutions and curricula. ... However, although steps for the structural standardization of medical education institutions have been discussed, this work has not yet begun. We believe that we should improve medical education institutions’ quality not by placing blame on new medical education institutions for being structurally inadequate; instead, we should compel them to take improvement measures. ... Although there are official regulations for establishing a university in Korea, these usually are minimum standards. Furthermore, there is no official regulation that accounts for the specific characteristics of medical schools.

However, no concrete steps were immediately made by the medical community to launch an accreditation project for medical schools in response to that article. Meanwhile, the Korea University Education Council, which had been conducting university-level evaluations, decided to conduct evaluations of specific academic fields. In 1996, the Korea University Education Council conducted evaluations of health-related universities, including medical schools, which led to severe concerns and complaints among the medical education community. Specifically, because of their lack of medical education expertise, some coercive evaluators focused mainly on external appearances such as facilities and equipment.

Furthermore, the evaluators tried to rank schools according to their assessment scores to present certain medical schools to the public as exemplars of excellence. At the end of his presidential term, President Kim Young-Sam’s civilian government advocated establishing several medical schools by implementing “approval first, facilities later” policies for establishing medical schools. Adherence to the principles of regional equilibrium in development and regulatory reform supported these policies. As the situation unfolded, the medical school certification project by autonomous organizations became an urgent issue that could no longer be postponed [2].

As a result, at the end of 1997, the Professional Committee for Accrediting New Medical Schools was formed. Research and development of the concepts and definition of medical education accreditation, including its definition, objectives, areas, standards, cycles, and procedures, were conducted. Finally, the Accreditation Board for Medical Education of Korea (ABMEK) was established in July 1998. In 1999, the ABMEK conducted preliminary accreditation for 8 new medical schools. The accreditation standards and systems were verified through this trial, and a full-scale medical education accreditation project began in 2000.

## Development of accreditation project

The ABMEK was reorganized into the Korean Institute of Medical Education and Evaluation (KIMEE) in 2003. The medical education accreditation projects from 2000 have been the 1st cycle (2000–2005), the 2nd cycle (2007–2010), the post-2nd cycle (2012–2018), and the current Accreditation Standards of the KIMEE 2019 (ASK2019, 2019–present) [3].

In the 1st cycle of accreditation, an emphasis was placed on preventing disruptions in new medical schools and achieving structural standardization of medical schools. In other words, the focus was on securing the minimum educational conditions and curriculum to fulfill the social accountability of nurturing doctors. Quantitative standards such as lecture rooms, laboratories, clinical training hospitals, basic educational facilities, materials for education, staffing of basic and clinical professors, and the curriculum’s basic composition were the most important criteria.

In the 2nd cycle of accreditation, which started in 2007, the goal was to go a step further and to develop medical education in Korea to reach international standards. Excellent standards were established in addition to existing mandatory and recommended standards. To this end, the KIMEE has benchmarked the accreditation standards, methods, and procedures of experienced countries such as the United States, the United Kingdom, and Australia, while reducing the number of quantitative criteria and significantly increasing the number of qualitative criteria for judging the validity of the content of medical education.

In the post-2nd cycle of the accreditation, which started in 2012, numerous quantitative criteria were changed to qualitative criteria. Each medical school was recommended to adopt a performance-based curriculum. Furthermore, by eliminating the cycle concept, each medical school’s self-assessment became a routine and regular activity. A particular emphasis was placed on character education, student safety, and ensuring personal rights. The Higher Education Act and the Medical Service Act were revised to adopt mandatory accreditation as a result of the ongoing efforts of the KIMEE at this time. As a result, accreditation became mandatory in the medical field, and a legal basis was established to close non-accredited universities. The KIMEE gained practical power, and finally, a medical school that failed accreditation multiple times was closed and an insolvent medical school was succeeded by other competent operating bodies.

In September 2016, the KIMEE was recognized as an accreditation agency representing Korea by the World Federation of Medical Education (WFME). ASK2019, which was modified to ensure that medical education accreditation standards were suitable for Korea’s present situation, was developed and applied starting in 2019. ASK2019 was designed to upgrade the medical education in Korea to the international level by emphasizing a learner-centered curriculum and integrating the 3 stages of education (basic medical education, graduate medical education, and continuing professional education). It also reflected the medical needs of society as a whole. The changed standards pursued by ASK2019 are indicators that will be used to assess the accreditation of medical schools in Korea in the future.

## The future of medical education accreditation in Korea

The accreditation project of the KIMEE has made outstanding contributions to medical education in Korea in the past 20 years. By setting minimum standards for the establishment and operation of medical schools, the proliferation of an excessive number of new medical schools was prevented. Furthermore, non-accredited schools were closed or handed over to other competent bodies. The accreditation standards of the KIMEE promoted the adoption of innovative curricula such as integrated education, performance-based education, and problem-based learning. It created a desirable environment for medical students to study and develop their skills through appropriate curricula. The concepts of medical humanities and social medicine were established according to KIMEE’s standards. Pursuing the excellence of basic medical education and responding well to globalization have been achievements of the KIMEE-led accreditation program. Professor George Miller said that “assessment drives learning,” but it is possible to say that “accreditation drives medical colleges” in Korea.

What should the accreditation of medical education look like in the future? In what direction should the future of medical education accreditation in Korea progress? In order to discuss future medical school accreditation projects, it is necessary to determine what medical education should look like in the future and what directions it should pursue. Topics often mentioned in discussions of the future of medical education include globalization, interprofessional education, health systems science, and the concept of a continuum of medical education. Harden [4] emphasized the globalization of medical education in a paper published in 2006. He predicted that medical education in the future will be forced to move in the direction of globalization due to the following facilitating factors: (1) The health care system is gradually globalizing. (2) Governments are under increasingly strong pressure to nurture medical personnel due to international competitiveness and societal demands. (3) International communication and exchange related to medical education are becoming more active than ever.

An international consensus on the philosophy and methods of medical education is being established, and many countries have adopted performance-based education systems. Therefore, it is necessary for future accreditation standards to verify whether appropriate education is being administered to train physicians who can act globally. In 2017, experts in medical education accreditation from Korea, Japan, and Taiwan argued that future medical school accreditation should be conducted following the concept of glocalization [5]. In other words, standards such as those of the WFME, Liaison Committee on Medical Education, and General Medical Council should be used as the basic framework. However, they should be modified and applied according to each country’s situation. Consideration should be given to dividing the accreditation system into separate systems: one based on local standards and the other based on global standards.

Along with globalization, inter-professional education is an essential factor in the future of medical education. Physicians, nurses, and many other health professionals will need to work together as a team. Starting in basic medical education courses, providing education on interprofessional communication will improve health professionals’ understanding of each other’s work, motivate teamwork, and foster cooperation skills. Future accreditation standards should therefore include such inter-professional education as an essential element. It is necessary to actively consider the participation of experts in other occupations such as nursing, dentistry, and pharmacy as medical education accreditation evaluators.

Health systems science, which has recently become the third domain of medicine, following basic medicine and clinical medicine, should also be emphasized. This field includes several areas such as professionalism, communication, leadership, value-based medicine, and patient safety. Humanities and social medicine were included as essential criteria in past accreditation cycles. It is also necessary to make health system science education an essential part of medical education accreditation in the future [6].

Future accreditation should ensure that the entire process of medical education―from basic medical education to postgraduate medical education and continuing professional education―can be conducted as an uninterrupted continuum. Currently, medical education accreditation is limited to basic medical education. The accreditation of postgraduate education and continuing education is currently operated without any communication with the KIMEE. This reality in Korea underscores the importance of the continuity and connection of all stages of medical education. According to international trends, we should integrate this segmented accreditation system, so that the boundary between basic medical education and postgraduate medical education will gradually be blurred [7]. Lastly, changes in the institutions being assessed are more important than changes in the accreditation body. The primary purpose of accreditation is not merely to check medical schools every few years to determine whether medical education programs meet the appropriate standards. Instead, it is necessary to ensure that medical education is the most important responsibility of medical schools. It is crucial that the operating personnel of medical schools—professors, students, and staff—participate in self-assessments to continue improving.

## Conclusion

Medical education accreditation should play a role in establishing and driving medical education in Korea, as it has done so far. It is crucial to look ahead to the directions in which medical education should move forward and to establish future directions of accreditation. The most crucial point is to perceive and respond proactively to new medical education trends worldwide, such as the globalization of medical education, the strengthening of inter-professional education, the emergence of health systems science, and the strengthening of continuity across education levels.

## Supplementary materials

Supplement 1. The audio recording of the abstract.
